# George Harley, F.R.S.

**Published:** 1899-12

**Authors:** 


**George Harley, F.R.S.** Edited by Mrs. ALEC TWEEDIE. Pp. xii., 360. London: The Scientific Press, Limited. 1899.

The life of Dr. George Harley was no ordinary one; he was a pioneer in various branches of scientific work. The story of his life and work is told by his daughter, and a most interesting volume has thus been given to the world. Her hero appears naturally as a wonderful man, learned in a variety of subjects—*nihil tetigit quod non ornavit*—nevertheless he was a somewhat disappointed man, inasmuch as "success and failure lie not in what a person has done, but in what he hoped to do." As a physician he was twice a partial success, as a man of science he was always in the front: he had, however, many difficulties to overcome; ill health overtook him when still quite young, and all his hopes in life were blasted by a severe attack of glaucoma, which necessitated complete retirement from work for a period of over two years. He was a man of many sides, and "hobbies were his solace and his joy." These hobbies are the points by which he will be usually remembered, but they must not make us forget the solid foundation of scientific knowledge on which his work was built.

As a charming companion, a mine of curious information on many out-of-the-way subjects, an excellent story-teller, and a ready speaker, he had a personality which greatly endeared him to a large circle of friends and patients: to those, and to others, the book is one which will give much pleasure, inasmuch as it can be characterised as a biographical and literary success.

